# Insights from terrain influence on gamma dose rate variability in lower Shivalik Himalayas

**DOI:** 10.1038/s41598-025-12124-1

**Published:** 2025-10-06

**Authors:** Prakhar Singh, Ankur Kumar, O. P. Nautiyal, Sandeep Singh, Devendra Singh

**Affiliations:** 1 Department of Applied Science (Physics), FET, Gurukul Kangri (Deemed to be University), Haridwar, Uttarakhand India 249404; 2 Department of Physics, Sri Dev Suman Uttarakhand University Gopeshwar, Chamoli, India Uttarakhand 246401; 3 Uttarakhand State Council for Science and Technology (UCOST), Vigyan Dham, Jhajhra, Dehradun, Uttarakhand India 248007

**Keywords:** Indoor environment, Ionizing radiation, Radiation dose, Gamma dose rate, Specific activity, Environmental sciences, Natural hazards

## Abstract

This study focused on assessing how natural background radiation levels vary due to spontaneous gamma emissions from the terrain of the Haridwar district. Gamma radiation levels are influenced by a mix of environmental and structural factors. Outdoor radiation is closely connected to soil composition and geology, while indoor radiation is affected by both external conditions and the characteristics of building materials. The range of observed indoor dose rates has been found from 0.07 to 0.31 µSv/h with an average value of 0.15 µSv/h, and outdoor dose rates from 0.06 to 0.28 µSv/h with an average of 0.13 µSv/h. The terrain of the administrative map of Haridwar district has been classified into two categories based on altitude, viz., Highland and Lowland. The results show that unexpectedly high radiation in certain areas and subtle variations between highlands and lowlands suggest unmodeled factors may be contributing to radiation exposure patterns. Indoor spaces showed higher gamma radiation compared to outdoors. Radiation exposure changes depending on age, with infants receiving the most. The interplay of potential outliers has been discussed in detail.

## Introduction

Radiation is a component of our natural environment, and it exists in a variety of forms, including gamma radiation. Gamma radiation is a form of electromagnetic radiation with a high energy level that is prevalent naturally. Our bodies have evolved to withstand certain levels of natural gamma radiation, but continuous exposure to excessive levels can be hazardous^1^. Earth and space are the primary sources of natural gamma radiation. Some radioactive elements, such as uranium, thorium, and potassium-40, can be found in the Earth’s crust^2,3^. Gamma rays are produced as a byproduct of the breakdown of certain elements over time. Additionally, cosmic radiation from outer space bombards our planet and contributes to the overall natural background radiation^4^. Another major contributor to background gamma radiation is radon (^222^Rn), a radioactive gas produced when radium in the Earth’s crust decays. High-energy gamma rays can easily pass through almost anything, including live tissue. Although live beings have some resistance to non-ionizing radiation, they are sensitive to the radiation emitted by radioactive substances. Gamma rays have the potential to cause cellular harm in humans due to their ability to ionize atoms and molecules within living cells^5,6^. High doses of gamma radiation can result in acute health effects, including radiation sickness, DNA damage, and an increased risk of cancer^1,7^. The natural gamma radiation to which humans are generally exposed is, however, quite low and poses only a little health risk. Low-level radiation exposure is generally harmless to human cells, and our systems have evolved methods to repair radiation-induced damage^8–10^. Understanding the dose of gamma radiation to people is of the utmost importance, involving a complex interplay of scientific research, industrial uses, and essential health concerns. By investigating its origins, qualities, and interactions with biological systems, it needs to comprehend the complexities of this irresistible force and its effects on human health and well-being. Whether arising from natural cosmic processes or human activities such as nuclear reactions and particle interactions, each source contributes to the intricate tapestry of radiation exposure^3,11^. To appreciate the possible hazards connected with gamma radiation, it is essential to differentiate between background levels and higher doses caused by anthropogenic activity. As one explores deeper into the mechanisms that control the interaction of gamma rays with live creatures, one discovers the intricacies of radiation biophysics and the creation of ionizing free radicals in biological tissues. Understanding the cascade impacts of these ionizations, ranging from cellular damage to potential genetic mutations, reveals the need to define allowable dosage limits for protecting human health^6,12^. A crucial aspect of this study involves the quantification and assessment of gamma radiation dose. The annual effective dosage from natural background radiation is around 2.4 mSv on a worldwide scale (millisieverts) where 0.8 mSv/y is due to terrestrial gamma exposure^7^. This dosage consists of cosmic radiation, radiation from terrestrial sources, and radon. The recommended dose limit for occupational exposure to natural gamma radiation is often greater than for the general population, as particular vocations may need persons to operate in locations with elevated radiation levels. Notably, the suggested dosage limits are far lower than the levels at which acute adverse health consequences are reported^13,14^. They are established with a large margin of safety to account for any differences in sensitivity among individuals and to limit the risks of radiation-induced health consequences over the long term. This study assesses the methodology applied in dosimetry, precision equipment, and international standards that support global radiation protection systems. By following tight dosage monitoring and safety measures, society strives to minimize any harm and protect those exposed to gamma radiation^15^.

Primarily, naturally occurring ^40^K decays spontaneously by emitting a gamma photon of 1.465 MeV. The decay chains of Radium-226 (^226^Ra) and Radon-222 (^222^Rn), two prominent radioactive isotopes that primarily undergo alpha decay, resulting in the transmutation of ^226^Ra into ^222^Rn, which subsequently decays into Polonium-218 (^218^Po). Additionally, both isotopes are associated with gamma emissions, characterized by the releasing photons of energies of 186 keV and 510 keV. Although it is important to note that the precise energies and intensities of these emissions can vary depending on the decay pathway.

The present study in the Haridwar region is driven by the imperative to systematically evaluate environmental radiation levels, given its distinct geological characteristics and significant population density. By conducting a radiological gamma survey and quantifying the activity concentrations of radionuclides such as ^226^Ra, ^232^Th, and ^40^K, this research aims to elucidate the spatial distribution of radiation exposure. Estimating the terrestrial radiological dose to humans is essential for assessing potential health risks and ensuring public safety. The findings will provide critical insights for local authorities, facilitating the development of informed radiation protection strategies.

## Methodology

### Study area

**Fig. 1:**
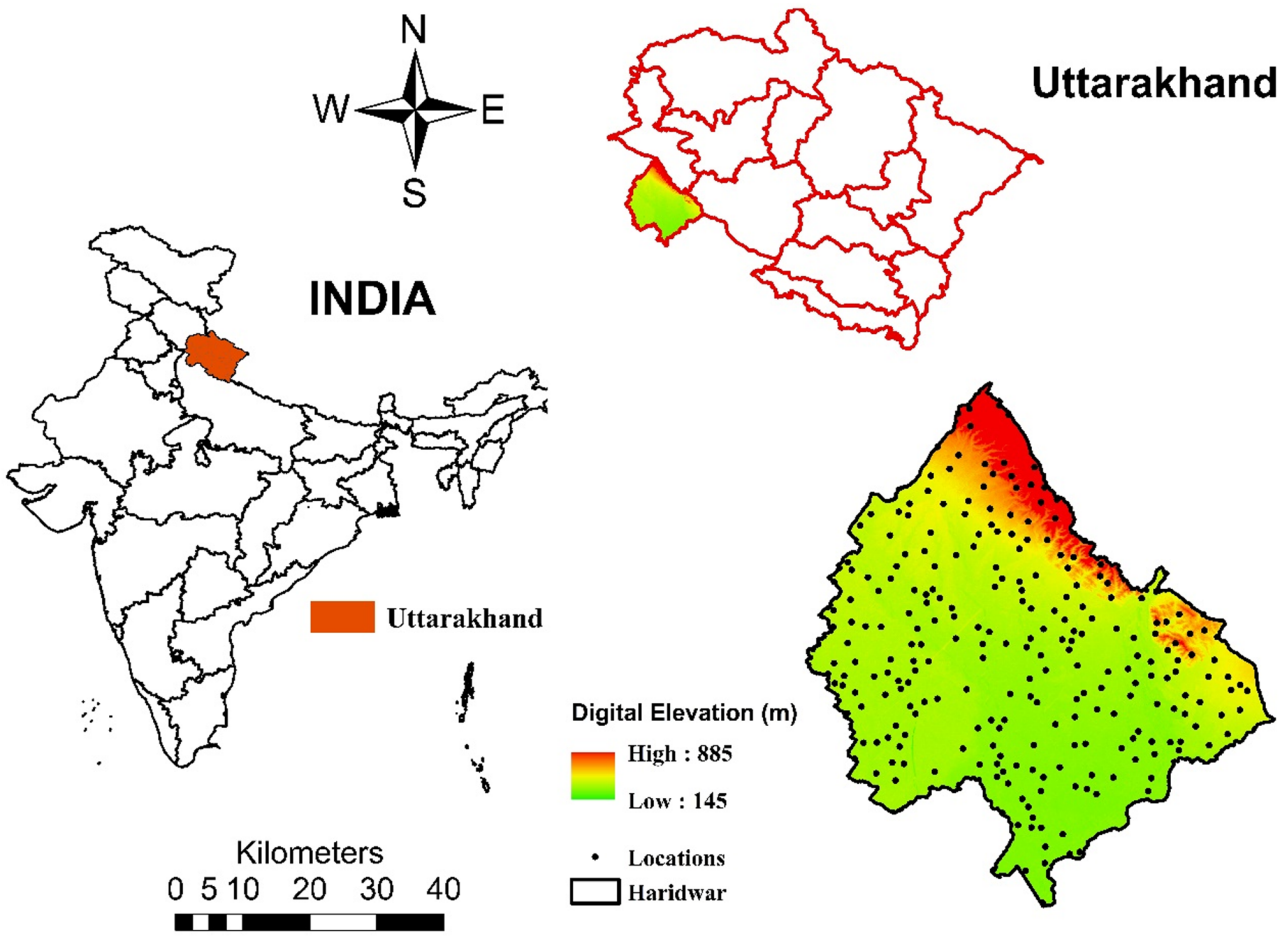
Digital Elevation Model (DEM) of Haridwar district, Uttarakhand, India (inset), with sampling locations. Elevation ranges from 145 m (green) to 885 m (red).

The present study has been performed within the administrative boundary of Haridwar district of Uttarakhand. Figure 1 depicts the digital elevation model (DEM) of Haridwar district of Uttarakhand state of India. All maps have been generated using ‘QGIS-3.40.6 Bratislava LTR’ which is an open-source geographic information system (GIS) software under GNU general public license version 2 (1991)^16^. This data has been downloaded from USGS Earth Explorer under OpenGL 4.0 license. This DEM dataset (AST14DEM) is a product of ASTER GDEM (Advanced Spaceborne Thermal Emission and Reflection Radiometer Global Digital Elevation Model) version 3.0 which is improvised over previous ASTER models^17^. The digital elevation is calculated in meters (*m*). This map consists of a colour gradient to represent elevation, ranging from green (low elevation, 145 m) to red (high elevation, 885 m). It also shows numerous black dots, labelled as “Locations,” which likely represent sampling points or specific sites of gamma dose measurements within the study area. At each location, indoor and outdoor gamma dose rates have been measured, with the indoor measurement taken inside a residential building and the outdoor measurement taken outside and away from the building. Haridwar consists of residential city land, agricultural land and reserved forest in the foothills of the Shivalik range of the great Indian Himalayas. The present study has been conducted by measuring ambient gamma dose rates at random locations without any bias.

### Field measurement

The ambient dose equivalent and dose equivalent rate for gamma radiation have been measured in the study area before sampling, utilizing an ATOMTEX-AT6130C pocket dosimeter. Assessing background gamma radiation levels constitutes a fundamental aspect of terrestrial radiation studies within a specific geographical region. This device operates on the principle of count rate using a Geiger-Muller tube in the presence of X-rays and gamma radiation. The count rate is then converted into dose rates within a specific energy range. Additionally, this device is also embedded with energy-compensating filters for the correction of sensitivity within the given energy range. It covers a broad measurement range of 0.1 µSv/h to 100 mSv/h (equivalent to 10 µR/h to 10 R/h), across an energy range spanning 50 keV to 3 MeV. Notable features include its quick response time, customizable audible and visual alarms that trigger when preset radiation thresholds are surpassed, and a clear, backlit digital display for effortless reading. The AT6130C boasts a sensitivity of 0.6 pulses per second per µSv/h for ^137^Cs and a stated relative intrinsic error of no more than ± 20%. The present study comprises of measurement of ambient gamma dose rate (µSv/h) in 250 random locations within the administrative boundary of Haridwar district at 5% uncertainty above 1 m surface of the ground. The measurement of gamma dose rates has been carried out in the month of December, when the meteorological conditions, characterized by low humidity, stable temperatures, and minimal rainfall, have been favourable for obtaining consistent and reliable data.

### Soil sampling for outliers

In the present study, radioelemental analysis was conducted on soil samples collected from six locations identified as outliers, as well as from five randomly selected locations corresponding to gamma dose rate measurement sites. The five random soil samples were collected to serve as representative controls for background comparison, enabling a more robust assessment of radionuclide variation across the study area and providing a baseline against which the outlier samples could be evaluated. The soil samples were obtained from outdoor environments and analysed for their radionuclide content using gamma spectrometry. The spectral data corresponding to various gamma emissions from the radionuclides in soil samples have been acquired using the Atomtex AT1315 gamma-beta spectrometer.

### Radiometric analysis

The soil samples were dried, crushed, and sieved to a 150 μm size in a plastic container for approximately one month, until a secular equilibrium had been achieved between the radionuclides and their progeny. The specific activity (Bq/kg) of naturally occurring radionuclides (^226^Ra, ^232^Th, and ^40^K) have been estimated using Atomtex-AT1315 gamma spectrometer, which consists of NaI(Tl) 63 × 63 mm coaxial scintillation detector with 1024 ADC channels which has a constant relative efficiency of 40% as well as a resolution at full-width half maximum (FWHM) of 2 keV at the 1332 keV gamma line at ^60^Co. A crystal diameter of 63 mm was operated under high voltage with a bias of + 3000 V (DC). The detector had been covered with a 12 cm thick lead shield to minimize the background radioactivity from its surrounding environment. The detection energy range of this spectrometer is 50 keV-3 MeV of gamma radiation with typical energy resolution at 662 keV (^137^Cs) by 8% and a limit of activity measurement intrinsic relative error to be 20%. The statistical counting of gamma energies has been continued for each sample for up to 6 h to get a resolved spectrum.

### Mathematical Estimation of dose and health risk

The dose of terrestrial gamma radiation is estimated in terms of annual effective dose (AED) for indoor and outdoor occupancy as follows^18–20^:

1 $${AED}_{in}\left(\mu\:Sv/y\right)={D}_{in}\times\:{T}_{Exp}\times\:DCF\times\:{O}_{in}$$

2 $${AED}_{out}\left(\mu\:Sv/y\right)={D}_{out}\times\:{T}_{Exp}\times\:DCF\times\:{O}_{out}$$

where D_in_ and D_out_ are measured gamma dose rates in indoors and outdoors, respectively. DCF is the dose conversion factor derived using the biokinetic model of ICRP, i.e., 0.7 Sv/Gy for indoors and outdoors. T(hours) is the time spent in the year of total exposure, i.e., 8760 h/y. O is the occupancy factor, i.e., 0.8 for indoors and 0.2 for outdoors^12^.

Another parameter is evaluated to quantify the risk factor of the exposure to gamma radiation, namely Lifetime Effective Dose (LED), which is calculated for the duration of life as follows^7^:

3 $$\:LED=\left[52{\times\:A}_{Adult}+16\times\:{A}_{Children}+2\times\:{A}_{Infant}\right]$$

where A represents the age-dependent annual effective dose for infancy, adolescence, and adult ageing phases given by Eqs. (1) and (2). The dose conversion factors for infants, adolescents, and adults are 0.9, 0.8, and 0.7, respectively.

The probability of cancer occurrence in a population over a given lifespan is a measure of cancer risk. The excess lifetime cancer risk (ELCR) has been determined by multiplying the values of the LED and RF, or lifetime effective dosage and risk factor, respectively. The ICRP 60 risk factor has an associated value of 0.05 in the general population. The ELCR is evaluated as follows^21^:

4 $$\:ELCR=LED\times\:RF$$

## Result and discussion

Table 1 summarizes the statistical parameters of indoor and outdoor gamma dose rates, their ratio, and the altitude profile of study locations. Analysis of these parameters reveals interesting insights into the relationship between these variables and the factors that influence terrestrial gamma radiation levels.

The average indoor gamma dose rate of 0.15 µSv/h is slightly higher than the average outdoor gamma dose rate of 0.13 µSv/h, resulting in an average indoor-by-outdoor ratio of 1.16. The indoor gamma dose rate variability depends upon the abundance of naturally occurring radioactive materials (NORMs) present in building materials such as bricks, cement, sand, etc. Additionally, the ventilation rate of indoor environments plays a substantial role in modulating radiation exposure levels by influencing the accumulation of radioactive gases, such as radon (^222^Rn) and thoron (^220^Rn) and their short-lived decay products, e.g., ^214^Pb and ^214^Bi. Poorly ventilated spaces tend to retain higher concentrations of these gases, leading to elevated indoor gamma dose rates due to the presence of their gamma-emitting progenies. The range of observed values is significant, with indoor dose rates ranging from 0.07 to 0.31 µSv/h and outdoor dose rates from 0.06 to 0.28 µSv/h. The minimum altitude is 216, and the maximum altitude is 813. The standard deviation arises up to 0.05 µSv/h in both the data, indicating a moderate spread of data around their respective means. The mode of the indoor dose rate (0.14 µSv/h) is also greater than the outdoor dose rate (0.11 µSv/h), indicating its natural tendency in the natural environment. Skewness values for outdoor (1.37) and indoor (1.28) are positive, implying that the distribution of both indoor and outdoor gamma dose rates is skewed to the right, with a longer tail towards higher values. Kurtosis values greater than 1 for both outdoor (1.17) and indoor (1.26) suggest a relatively peaked distribution compared to a normal distribution.

**Table 1:** Statistical parameters of altitude, ambient outdoor and indoor gamma dose rates (µSv/h), and indoor-to-outdoor ratio.

Radiological parametrs		Statistical parameters								
		Minimum	Maximum	Arithmetic mean	Standard deviation	Geometric mean	Median	Mode	Skewness	Kurtosis
Altitude		216	813	303.15	102.15	291.36	269	229	2.65	7.55
Gamma dose rate (µSv/h)	Outdoor	0.06	0.28	0.13	0.05	0.13	0.12	0.11	1.37	1.17
	Indoor	0.07	0.31	0.15	0.05	0.14	0.14	0.14	1.29	1.26
Indoor-to-outdoor ratio		0.6	1.9	1.16	0.18	1.15	1.1	1.1	0.72	1.77
Annual effective dose (mSv/y)	Indoor	0.34	1.52	0.74	0.26	0.7	0.69	0.69	1.29	1.26
	Outdoor	0.07	0.34	0.16	0.06	0.15	0.15	0.13	1.37	1.17
	Total	0.42	1.86	0.9	0.31	0.86	0.84	0.72	1.34	1.33
Age-dependent annual effective dose (mSv/y)	Infant	0.54	2.4	1.16	0.4	1.1	1.08	0.93	1.34	1.33
	Children	0.48	2.13	1.03	0.36	0.98	0.96	0.83	1.34	1.33
	Adult	0.42	1.86	0.9	0.31	0.86	0.84	0.72	1.34	1.33
LED (mSv)		30.38	135.82	65.71	22.79	62.41	61.21	52.72	1.34	1.33
ELCR (mSv)		1.52	6.79	3.29	1.14	3.12	3.06	2.64	1.34	1.33

The observed range of gamma dose rates likely reflects variations in the local geology and the presence of naturally occurring radioactive materials (NORMs), such as radium (^226^Ra), thorium (^232^Th), and potassium ^40^K), in the soil and rocks. Areas with higher concentrations of these radionuclides will generally exhibit higher gamma dose rates. A ratio less than 1 could imply a higher outdoor dose rate, potentially due to the presence of radioactive materials in the soil. The positive skewness of both indoor and outdoor dose rate distributions suggests that areas with significantly higher gamma radiation levels, possibly due to localized geological anomalies or specific building materials, are less common but still contribute to the overall distribution. The kurtosis values indicate a higher probability of observing values closer to the mean, with a rapid drop-off in probability for values further away from the mean when compared to a normal distribution.

### Annual effective dose

The data of the Annual Effective Dose (AED) in Table 1 provides a detailed statistical summary of radiological exposure, capturing indoor, outdoor, and total AED values in millisieverts per year (mSv/y). Indoor AED ranges from 0.34 to 1.52 mSv/y, with a mean of 0.74 mSv/y and a standard deviation of 0.26 mSv/y, indicating moderate variability in indoor exposure. The geometric mean and median, both approximately 0.7 mSv/y, closely align, reflecting a fairly symmetrical distribution. Outdoor AED values are lower, ranging between 0.07 and 0.34 mSv/y, with a mean of 0.16 mSv/y and a standard deviation of 0.06 mSv/y. This lower mean highlights reduced radiological exposure in outdoor environments due to dispersion and lower accumulation compared to enclosed spaces. Total AED combines indoor and outdoor exposure, with a range of 0.42 to 1.86 mSv/y, a mean of 0.9 mSv/y, and a standard deviation of 0.31 mSv/y, showcasing an integrated perspective on radiological exposure. Furthermore, the data shows a clear trend of decreasing annual effective dose with increasing age. Infants receive the highest average dose (1.16 mSv/y), followed by children (1.03 mSv/y), and then adults (0.9 mSv/y).

The median (0.86 mSv/y) and mode (0.72 mSv/y) are both lower than the mean for the annual effective dose for adults, suggesting a right-skewed distribution with some individuals experiencing significantly higher doses than the average. This skewness is further confirmed by the skewness value of 1.34, indicating a positive skew. The kurtosis value of 1.33 indicates a leptokurtic distribution, meaning the data has heavier tails and a sharper peak compared to a normal distribution. This suggests a higher probability of extreme values, both low and high, in the dataset. These statistical parameters provide valuable insights into the distribution and characteristics of annual effective doses, highlighting the variability in exposure levels in the study area.

### LED and ELCR

The long-term implications of radiation exposure by examining Lifetime Effective Dose (LED) and Excess Lifetime Cancer Risk (ELCR). LED, representing the total accumulated radiation dose over a lifetime, averages 65.71 mSv, but with a significant range (30.38–135.82 mSv). Similarly, ELCR, which estimates the added probability of developing cancer from radiation, shows a range from 1.52 to 6.79, with an average of 3.29. This underscores the individual nature of cancer risk from radiation exposure^19^.

The substantial standard deviations for both LED (22.79 mSv) and ELCR (1.14) further emphasize this variability. The data’s distribution, skewed towards higher values, suggests that a smaller segment of the population may experience disproportionately significant LED and ELCR. The distribution also exhibits heavier tails than a normal distribution, indicating a greater likelihood of extreme values. This observation underscores the importance of personalized risk assessments that account for individual variations and potential outliers when evaluating the long-term consequences of radiation exposure. It is important to note that ELCR is a statistical estimate and does not predict the actual occurrence of cancer in any individual.

### Indoor to outdoor ratio

The exploration of the indoor-to-outdoor gamma dose ratio is a crucial area of research within the field of radiation exposure assessment. By dissecting this ratio, one can endeavour to unravel the multifaceted interplay between indoor environments, external surroundings, and their resulting radiation doses. The indoor-to-outdoor gamma dose rate ratio in the Haridwar district exhibited significant variability, ranging from 0.6 to 1.9, with an arithmetic mean of 1.16, a standard deviation of 0.18, and a geometric mean of 1.15. The median value was 1.10, and the distribution was moderately skewed (skewness: 0.72) with a kurtosis of 1.77, indicating a leptokurtic nature. These values suggest a consistent predominance of indoor gamma dose rates over outdoor levels across the majority of the surveyed locations. However, the range also indicates a few cases where outdoor levels surpassed indoor values, pointing to localized environmental influences. This ratio reflects localized micro-environmental conditions, suggesting that indoor environments consistently exhibit elevated dose rates relative to their outdoor counterparts, though in some instances, outdoor levels exceed indoor values. The discernment of this ratio stands as a cornerstone in devising meticulously tailored strategies encompassing radiation abatement, architectural innovations, and regulatory measures, all working harmoniously to curtail cumulative exposure and safeguard the holistic well-being of individuals situated in diverse environmental settings. Several studies have been conducted globally to monitor indoor-outdoor gamma dose to inhabitants of the locale as depicted in Table 2.

**Table 2:** Globally estimated indoor-outdoor gamma dose ratio.

Country/Region	Indoor–outdoor gamma dose ratio	References
Slovakia, South America	1.2	^22^
Slovenia	1.3	^23^
Netherland, Sweden	2	^7,24^
Hong Kong	2.3	^25^
Australia, Norway, France Bulgaria, Russia	1.1	^7^
Hungry, Iran, China	1.6	^22,26^
United Kingdom	1.8	^27^
Japan, Malaysia, Denmark, Finland	1.0	^6,7^
Lithuania, Poland, Ireland, Thailand	0.6	^7^
Germany, Belgium, Romania, Switzerland, Bhilai India	1.4	^7,28^
United States, Iceland	0.8	^7^
Rural and drug district of India	1.3	^29,30^
Gobi area South India	1.1	^31^
Village of Telangana	1.1	^32^
Balod District of India	1.4	^19^
World Population Weighted Average	1.4	^7^

### Statistical distribution

**Fig. 2:**
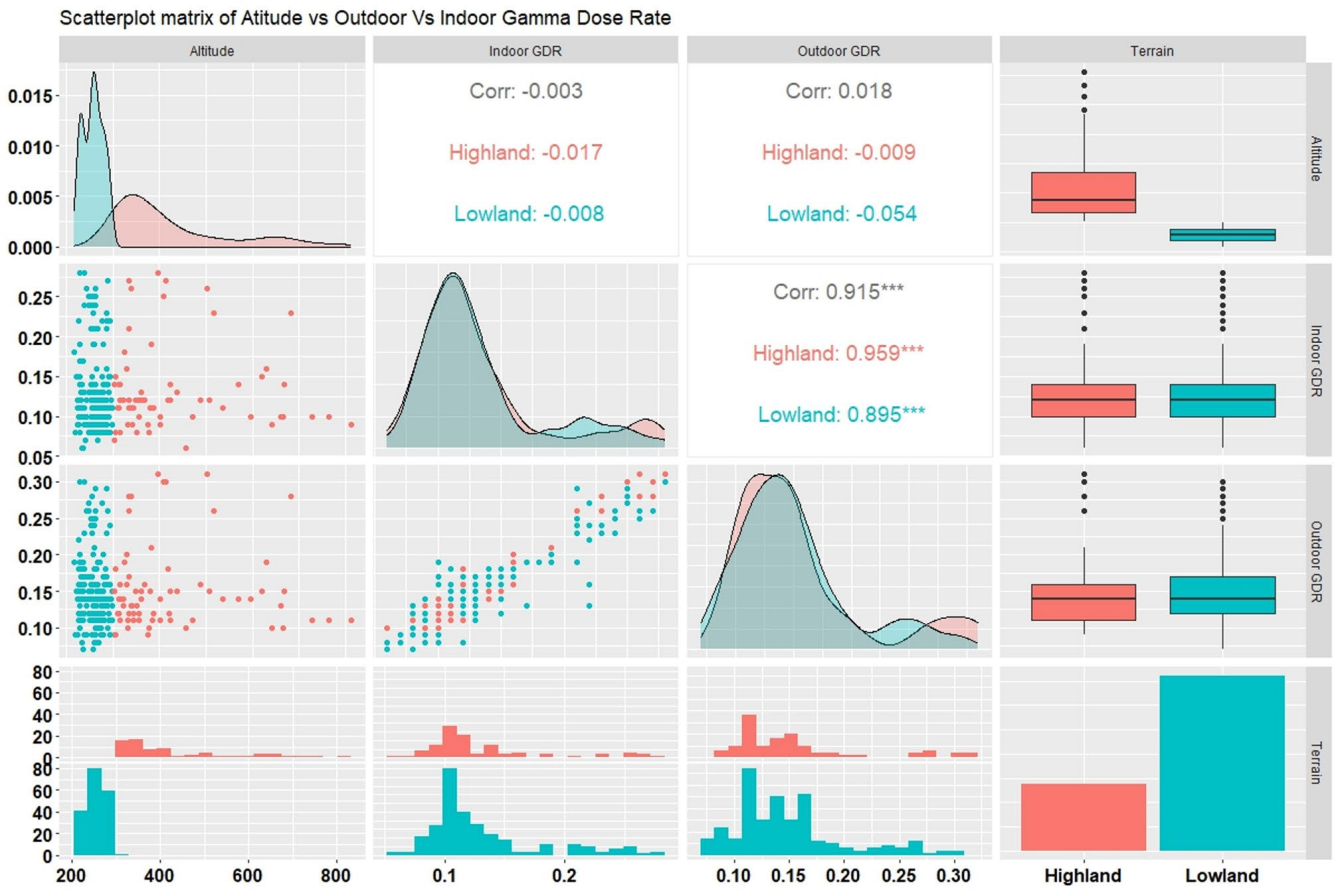
Scatterplot matrix exploring correlations between altitude, indoor and outdoor gamma dose rates (GDR), and terrain type (highland/lowland) in Haridwar. Includes density plots, scatterplots, correlation coefficients, and boxplots.

Haridwar district contains the Ganga-river basin and alluvial plains majorly in addition to a small sector of the lower range of Shivalik hills. Therefore, the present analysis segregates between these two terrains, namely lowland with an altitude below 300 m above mean sea level and highland with above 300 m of altitude. The collected dataset has been examined statistically against this segregation and attempted to be analysed through a bimodal approach. The analysis regards the statistical output, which is illustrated by the scatterplot matrix in Fig. 2.

Scatterplot matrix displays relationships among univariate parameters, including altitude, indoor gamma dose rate (GDR), outdoor gamma dose rate (GDR), and terrain type (classified as either “Highland” or “Lowland”). Each panel in the matrix offers a visual or statistical representation of how these variables relate to one another, highlighting correlations, distributions, and variations. Scatterplots reveal patterns or trends in pairwise relationships, with colour-coding helping to distinguish between highland and lowland data.

#### Density distribution

The density plots in the scatterplot matrix provide a visual representation of the distribution of each variable, such as altitude, indoor gamma dose rate, and outdoor, within the dataset. These plots are presented on the diagonal of the matrix, offering insight into the frequency and spread of values for both highland and lowland terrains. The curves are colour-coded to distinguish between the two terrains, with red representing highland regions and blue representing lowland regions. For altitude, the density plot reveals a bimodal distribution, indicating two distinct groups corresponding to high and low elevations. This separation aligns with the terrain classification, as highlands occupy a range of higher altitudes, while lowlands are concentrated at lower elevations. The density curve for lowlands peaks sharply at lower altitudes, reflecting a narrower distribution, whereas the highlands exhibit a broader spread across a wider range of altitudes, suggesting more variability in elevation within this terrain.

The distribution for outdoor (µSv/h) shows a more pronounced separation between highland and lowland curves, with highland regions generally exhibiting higher gamma dose rates. This suggests that outdoor radiation levels are influenced by environmental factors such as soil composition or natural radioactive sources, which may be more prevalent in highland areas^33–35^. In contrast, the indoor (µSv/h) density plot shows greater overlap between the two terrains, though highlands still demonstrate slightly elevated levels on average. This overlap suggests that indoor radiation levels are partially influenced by outdoor conditions but may also depend on factors such as building materials, structural shielding, and indoor ventilation. Both indoor and outdoor (µSv/h) density plots exhibit unimodal distributions, indicating a central tendency in radiation levels for each terrain while maintaining differences in their overall ranges. Together, these density plots provide critical insights into the variability and central tendencies of the parameters across terrains, helping to understand how environmental and structural factors shape radiation patterns.

**Table 3:** Statistical summary parameters used in box plot representation of gamma dose rate measurements across lowland and Highland stratifications in the Haridwar district. The parameters include range, mean, standard deviation, median, skewness, quartile (Q1 and Q3), interquartile range (IQR), and variance, providing insights into the distribution and variability of gamma dose rates within each stratification.

Statistical Parameters	Lowland			Highland		
	Altitude (m)	Gamma dose rate (µSv/h)		Altitude (m)	Gamma dose rate (µSv/h)	
		Outdoor	Indoor		Outdoor	Indoor
Range	224–298	0.07–0.23	0.08–0.24	302–813	0.06–0.28	0.09–0.31
Mean	256.87	0.12	0.14	423.03	0.13	0.15
Standard deviation	19.85	0.03	0.03	129.78	0.05	0.06
Median	259	0.12	0.14	374	0.12	0.14
Skewness	− 0.07	1.4	0.64	1.36	1.49	1.5
First quartile	224	0.07	0.08	302	0.06	0.09
Second quartile	270	0.13	0.16	470	0.14	0.16
IQR	46	0.06	0.08	168	0.08	0.07
Variance	393.85	0.0010	0.0012	16843.38	0.0027	0.0034

#### Whisker’s box

The statistical evaluation of gamma dose rates across lowland and highland regions of Haridwar reveals notable variations, as shown in Table 3. The altitude in lowland areas ranged from 224 to 298 m, with a mean of 256.87 m, whereas the highland region spanned a broader range of 302–813 m, averaging 423.03 m. Gamma dose rates, both outdoor and indoor, exhibited slightly higher values in highland areas, with mean indoor and outdoor rates of 0.15 µSv/h and 0.13 µSv/h, respectively, compared to 0.14 µSv/h and 0.12 µSv/h in the lowlands. The skewness values indicate a right-skewed distribution, particularly in the highland data (skewness > 1 for both indoor and outdoor), suggesting a prevalence of lower values with a few higher outliers. In contrast, lowland gamma dose rates show moderate skewness, suggesting more symmetric distributions. The interquartile range (IQR) was greater in the highlands, reflecting greater variability in both altitude and dose rate. In the lowland region, standard deviations for outdoor and indoor gamma dose rates are relatively low (0.03 µSv/h each), with corresponding variances of 0.001 and 0.0012. This indicates a relatively uniform distribution of gamma dose rates in these areas, suggesting minimal environmental fluctuations and a more stable radiological profile. Conversely, in the highland region, the standard deviation is notably higher for both outdoor (0.05 µSv/h) and indoor (0.06 µSv/h) gamma dose rates, with variances of 0.0027 and 0.0034, respectively. This reflects greater variability and a wider spread of data points from the mean. The higher variability may be attributed to heterogeneous geological formations.

The boxplots presented in the scatterplot matrix (Fig. 2) visually summarize the distributions of altitude, indoor gamma dose rate (µSv/h), and outdoor gamma dose rate (µSv/h) across highland and lowland terrains. These visualizations complement the statistical metrics outlined in Table 3, highlighting differences in central tendency, spread, and outlier presence. As expected, highland regions exhibit significantly higher altitudes with a broader interquartile range (IQR), indicating greater variability in elevation. The presence of several high-altitude outliers further emphasizes the topographic diversity in these areas, while lowlands show a tighter altitude range and clustered median values at lower elevations. For indoor gamma dose rates, the highlands display a slightly higher median than the lowlands, though overlapping IQRs indicate comparable variability within both regions. Outdoor gamma dose rates reveal a clearer distinction: highlands not only have a higher median but also a wider IQR and more frequent outliers, suggesting greater spatial variability and potential radiation hotspots. In contrast, the lowland outdoor dose rates are more uniform, reflecting relatively consistent environmental conditions.

#### Histograms

The histograms displayed at the bottom of the scatterplot matrix provide detailed insights into the frequency distribution of the three key parameters, including altitude, indoor gamma dose rate (µSv/h), and outdoor (µSv/h) for both highland and lowland terrains. Each histogram represents the count of observations within specific ranges (bins) of the respective variables, helping to understand the overall distribution and the concentration of data points across the terrains. The histograms show a distinct bimodal pattern corresponding to highland and lowland regions. The lowland histogram (blue) is sharply concentrated in a narrow range at lower altitudes, with a high peak, indicating that most lowland observations are clustered around a specific elevation. In contrast, the highland histogram (red) is more spread out, with a broader range of altitudes and a flatter peak, suggesting greater variability in elevation.

The indoor gamma dose rate (µSv/h) histograms show overlapping but slightly distinct distributions for the two terrains. The lowland histogram (blue) is centred around lower gamma dose rates, with most values falling within a relatively narrow range. The highland histogram (red), on the other hand, shows a slight shift toward higher dose rates, with a more spread-out distribution, indicating greater variability in indoor radiation levels. This pattern suggests that indoor gamma radiation in highland regions may be influenced by environmental factors such as natural radiation sources, as well as differences in building materials.

The histograms show a clearer distinction of outdoor gamma dose rate (µSv/h) between highland and lowland terrains. The lowland histogram (blue) has a narrow peak at lower dose rates, reflecting a more uniform and lower overall radiation level outdoors. Conversely, the highland histogram (red) is broader and shifted toward higher dose rates, indicating that outdoor radiation levels in highland areas are generally higher and more variable. This disparity is likely due to differences in geological factors, such as the presence of radioactive elements in the soil and rock composition, as well as the reduced atmospheric shielding at higher altitudes.

#### Pair-wise correlation

The pairwise correlation coefficients in the scatterplot matrix reveal the relationships between altitude, indoor gamma dose rate, and outdoor, with distinctions drawn between highland and lowland terrains. Notably, a strong positive correlation (*r* = 0.91 overall) is observed between indoor and outdoor. This correlation is slightly stronger in highland areas (*r* = 0.96) compared to lowlands (*r* = 0.89), suggesting that outdoor environmental factors, such as natural radiation sources, have a more pronounced impact in elevated regions. In contrast, altitude shows weak or negligible correlations with both indoor (*r* = -0.003) and outdoor (*r* = 0.018), implying that altitude alone is not a direct determinant of gamma dose rates. However, terrain-specific correlations highlight subtle differences: highland areas exhibit slightly negative correlations between altitude and gamma dose rates, while lowlands show even weaker or insignificant associations. These findings suggest that while gamma radiation levels are predominantly governed by localized environmental and structural factors, the relationship between indoor and outdoor is consistent and strong across both terrains.

### Statistical fitting

**Fig. 3:**
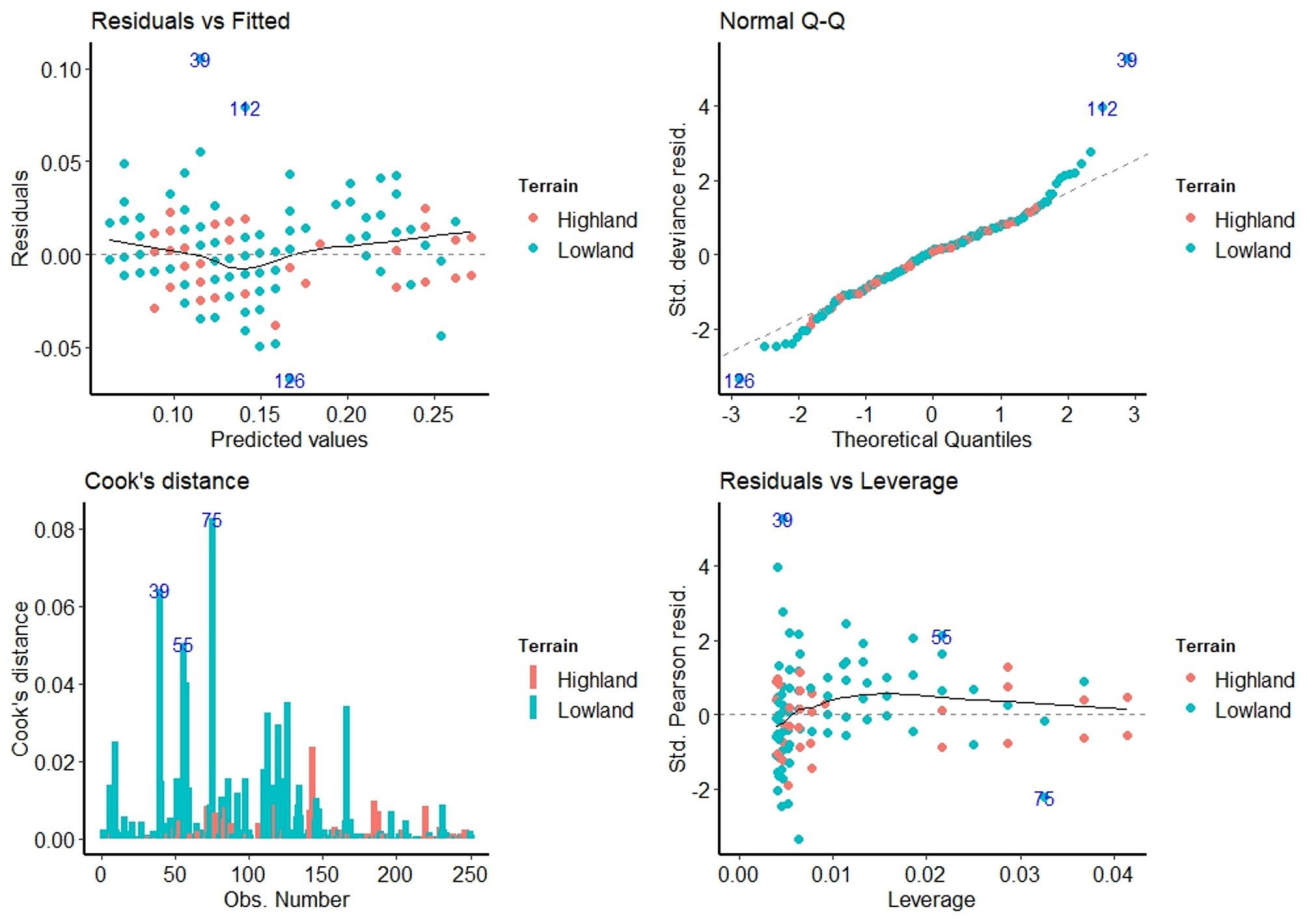
Model assumptions and influence: (1) Residuals versus Fitted shows slight non-linearity; (2) Normal Q–Q highlights deviations at the tails; (3) Cook’s Distance identifies influential lowland observations; and (4) Residuals versus Leverage flags points with high leverage and residuals. Highland (red) and lowland (blue) patterns are distinguished.

The dataset is inspected for statistical fitting to compare with standard distributions. Fig. 3 depicts the results of the fitting operations to regression models.

The diagram consists of four diagnostic plots commonly used in regression analysis: Residuals vs. Fitted, Normal Q–Q, Cook’s Distance, and Residuals vs. Leverage. These plots collectively assess the adequacy of a regression model by examining residual behaviour, the influence of observations, and model assumptions such as normality, linearity, and homoscedasticity. The parameters analysed here appear to involve terrain-based observations (highland and lowland), represented using distinct colours (red for highland and blue for lowland), aiding in the interpretation of data across these terrains.

#### Residuals after fitting

The assessment of a statistical model’s validity often begins with scrutinizing the underlying assumptions of linearity and homoscedasticity, both of which are crucial for the accurate interpretation of regression analysis. The Residuals vs. Fitted plot serves as a primary diagnostic tool in this evaluation, providing a visual representation of the relationship between the model’s predicted values and the residuals, and the discrepancies between observed and predicted outcomes. In the present analysis, the Residuals vs. Fitted plot reveals a generally flat trend in the smoothed line, suggesting that the linearity assumption is reasonably met. This flatness indicates that the model’s predictions do not systematically over- or underestimate the dependent variable across the range of fitted values, supporting the appropriateness of a linear model^36^. Furthermore, the plot’s examination of homoscedasticity, the assumption of constant variance of residuals, reveals a relatively consistent spread of data points around the smoothed line, implying that the variability in prediction errors is largely uniform across the range of predicted values. While a minor increase in variability might be present in the middle range, the overall pattern does not strongly suggest heteroscedasticity. The absence of a clear distinction between Highland and Lowland terrains in the distribution of residuals indicates that the model adequately accounts for the effect of this categorical variable. However, potential outliers, denoted by points 39, 112, and 126, necessitate further investigation, as these points exhibit relatively large residuals and could distort the model’s estimates^37,38^.

#### The Q–Q plot

The validity of many statistical inferences derived from regression analysis hinges on the assumption that the model’s residuals are normally distributed. The Normal Q–Q (Quantile–Quantile) plot offers a graphical means of assessing this crucial assumption by comparing the quantiles of the standardized residuals to the theoretical quantiles of a standard normal distribution^39^. In this case, the Normal Q–Q plot demonstrates that the majority of data points align closely with the diagonal reference line, indicating that the residuals largely conform to a normal distribution. However, a discernible deviation from the diagonal is observed in the tails of the distribution, particularly for higher positive quantiles, suggesting the presence of heavier tails than would be expected under perfect normality. This observation implies that extreme values might be slightly more prevalent than predicted by a normal distribution. Furthermore, points 39 and 112 stand out again. The absence of discernible clustering of points based on terrain (Highland or Lowland) suggests that the normality assumption holds relatively consistently across both categories. While the observed deviations from perfect normality are not severe, they nonetheless warrant consideration, particularly if subsequent analyses involve hypothesis testing or the construction of confidence intervals that rely heavily on the normality assumption. In such instances, exploring data transformations or employing robust statistical methods that are less sensitive to non-normality might be advisable^36^.

#### The Cook’s distance

A critical aspect of model diagnostics involves identifying data points that exert undue influence on the model’s estimated parameters. Cook’s distance provides a quantitative measure of this influence, with higher values indicating greater influence^40^. In the present analysis, the Cook’s Distance plot reveals that while no data points exceed commonly accepted thresholds for high influence (e.g., 0.5 or 1), observations 75, 39, and 55 exhibit relatively higher Cook’s distances compared to other observations. This suggests that these points have a somewhat stronger influence on the model’s coefficients, although not to an alarming degree. Interestingly, the plot also suggests a tendency for points associated with the Highland terrain to exhibit slightly higher Cook’s distances than those associated with the Lowland terrain. This observation could imply that the Highland data points, possibly due to factors not explicitly included in the model, have a greater degree of leverage in shaping the regression estimates. Although no points are excessively influential based on conventional cut-offs, a prudent approach would involve a closer examination of observations 75, 39, and 55 (Lowland). Investigating the characteristics of these data points, verifying the accuracy of their recorded values, and exploring potential reasons for their greater influence could provide valuable insights into the model’s behaviour and potentially lead to refinements that enhance its robustness^38^.

#### Leverage estimation

The Residuals vs. Leverage plot serves as a powerful tool for simultaneously assessing the leverage and residual magnitude of individual data points, thereby aiding in the identification of potential outliers that might exert undue influence on the model^41^. Leverage, in this context, quantifies the extent to which an observation’s predictor variable values deviate from the average values of the predictor variables. In the presented plot, the majority of data points exhibit low leverage, clustering towards the lower end of the x-axis, indicating that their predictor values are relatively close to the average. However, a few points, notably those associated with the Highland terrain, display somewhat higher leverage. Specifically, point 75 is closer to the 0.02 leverage line. This suggests that these observations have predictor values that are farther from the mean, potentially giving them greater influence on the slope of the regression line. Furthermore, the plot highlights point 39 and 55, both from the Lowland group, as having relatively high residuals combined with moderate leverage. These points are potential outliers that could be distorting the model’s estimates. The smoothed line in the plot does not deviate substantially from horizontality, suggesting no major issues with non-linearity. Although the absence of Cook’s distance contours makes it difficult to definitively assess the overall influence of these points, their positions in the plot warrant scrutiny^38,40^. The observation that Highland points tend to exhibit slightly higher leverage than Lowland points aligns with the findings from Cook’s Distance plot and suggests that the Highland terrain might be associated with greater variability in predictor values or other unmodeled factors^36^. These findings underscore the importance of thoroughly investigating the characteristics of data points with high leverage and large residuals to ensure the robustness and validity of the statistical model.

### Surface interpolation

**Fig. 4:**
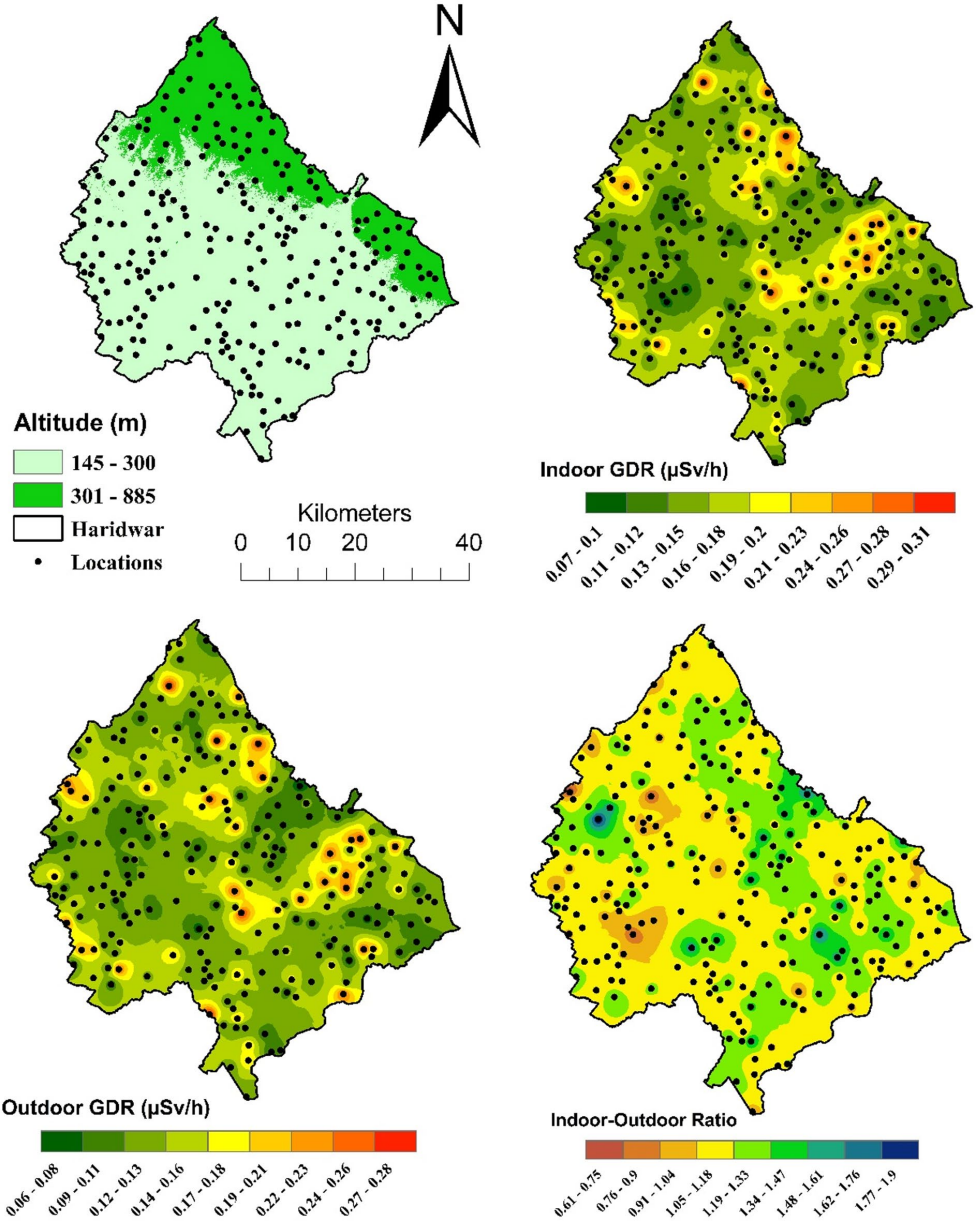
Maps of Haridwar district showing the spatial variation of altitude (m), indoor gamma dose rate (µSv/h), outdoor gamma dose rate (µSv/h), and the ratio between indoor and outdoor dose rates. Black dots represent measurement locations.

The acquired datasets have been interpolated over the map using ‘Inverse Distance Weightage (IDW)’ to analyse the spatial distribution of gamma radiation levels in indoor and outdoor environments across the Haridwar region and their relationship with geographical topography^42,43^. In Fig. 4, the maps collectively reveal spatial trends in environmental gamma radiation and their relationship with topographical features. Black dots represent sampling locations and ensure regional spatial coverage for a comprehensive assessment.

1. The top left map is an altitude map, illustrating the topographical variation across the Haridwar region, divided into two elevation ranges: 145–300 m and 301–885 m above sea level. The lighter green areas represent low-altitude zones (lowland), which dominate the region, while darker green regions signify higher-altitude areas (highland), concentrated in the northern and northeastern parts.
2. The top right map visualizes the spatial distribution of indoor gamma dose rates (µSv/h). Indoor GDR values range from 0.07 to 0.31 µSv/h, represented on a gradient scale where green indicates the lowest levels and red denotes the highest. In the lowlands, indoor GDR is generally lower, and more uniform compared to the highlands. This could be attributed to differences in building practices, with materials like concrete and brick, which may emit less gamma radiation than stone. Additionally, better ventilation in lowland homes may reduce the accumulation of radon gas indoors. Few localised hotspots are observed, indicating relatively stable indoor gamma radiation levels in lowland regions. In the highland areas, indoor GDR tends to be more concentrated and exhibits slightly higher variations compared to the lowlands. The map highlights localized indoor hotspots in highland regions, suggesting significant variability in radiation levels within residential settings.
3. The outdoor gamma dose rate map (bottom left) displays radiation levels in open environments, measured in the same units (µSv/h) as the indoor map. Values range from 0.05 to 0.34 µSv/h, with a gradient colour scheme from green (low levels) to red (high levels). Outdoor GDR in the highlands is generally lower compared to indoor GDR, but notable spatial variations still exist. Additionally, the thinner atmosphere at higher altitudes may allow slightly increased exposure to cosmic gamma radiation, adding to outdoor levels. However, the overall outdoor GDR remains relatively dispersed and less concentrated compared to indoor levels. The relatively stable terrain in the lowlands contributes to more uniform outdoor GDR levels, with fewer prominent hotspots.
4. The bottom right map represents the ratio of indoor to outdoor gamma dose rates, with values ranging from 0.61 to 1.90. The regions with low ratios, near 1, (green) indicate similar indoor and outdoor radiation levels, suggesting good ventilation or minimal influence from construction materials. High ratios (blue) indicate significantly higher indoor gamma dose rates compared to outdoor levels, often attributable to the use of radiation-emitting materials such as granite or concrete, as well as poor ventilation, which allows radon gas to accumulate indoors. Furthermore, most of the areas shows indoor to outdoor GDR ratio to be within the range of 1.05 (yellow) to 1.47 (green), except a few highlighted zones. It is established that the indoor gamma dose rate prominently exceeds the outdoor gamma dose rate, leaving a few outliers where outdoor values exceed the indoor ones. These sites have been observed as long-run agricultural lands. The soil samples have been collected to identify the dominating radionuclide in additional gamma dose rate within these lands. Furthermore, the majority of the areas exhibit an indoor-to-outdoor GDR ratio ranging from 1.05 (yellow) to 1.47 (green), with a few exceptions in highlighted zones. It is evident that the indoor gamma dose rate generally surpasses the outdoor gamma dose rate, aside from a few outliers where outdoor values are higher. These outlier locations have been identified as long-standing agricultural lands. Soil samples from these areas have been collected to determine the predominant radionuclides contributing to the elevated outdoor gamma dose rates.

### Outlier examinations

During the analysis of the indoor-to-outdoor gamma dose ratio (GDR), two significant outliers have been identified, representing extreme minimum and maximum values. These outliers correspond to sample numbers 39, 55, 56, 75, 112, and 126. Soil samples have been collected from the respective locations to explore the natural factors influencing these anomalies. Additionally, five supplementary samples have been obtained from randomly selected sites, where gamma dose rate measurements had previously been conducted. These sites are designated as locations 5, 10, 15, 20, and 25. The comprehensive datasets from this analysis, detailing the presence and concentration of radionuclides, are systematically presented in Table 4 for further examination.

**Table 4:** Table showing gamma dose rates, their ratio, and their corresponding specific activity in the collected soil samples.

Id	Locations	Altitude (m)	Gamma dose rate (µSv/h)		Ratio (Indoor/Outdoor)	Specific activity (Bq/Kg)		
			Outdoor	Indoor		^226^Ra	^232^Th	^40^K
39	Kalanjara Jadid	288	0.22	0.13	0.6	78.22	114.38	651.55
55	Tanda Banjara	288	0.27	0.26	1	65.88	98.21	872.38
56	Rithaura Grunt	229	0.1	0.1	1	74.86	107.68	420.37
75	Dhandera (CT)	286	0.21	0.29	1.4	89.43	132.26	682.75
112	Shahpur	287	0.22	0.16	0.7	72.38	86.87	621.47
126	HakimPur Turra	271	0.1	0.19	1.9	67.84	92.63	421.33
10	Daluwala Khurd	225	0.12	0.16	1.3	68.46	94.1	320.18
20	Sikraudha	276	0.11	0.12	1.1	74.38	109.12	408.64
30	Mukarrabpur	272	0.11	0.14	1.3	65.28	88.15	420.66
40	Sahpur Salhapur	267	0.21	0.19	0.9	59.12	78.38	710.49
50	Daluwala Mazbata	291	0.15	0.14	0.9	74.65	48.51	468.25

The table provides detailed data on various locations, focusing on altitude, gamma dose rates (both outdoor and indoor), the indoor/outdoor gamma dose rate ratio, and the specific activity of radionuclides (^226^Ra, ^232^Th, and ^40^K). Each location is identified by an ID and name, offering a comprehensive view of the radiological and environmental characteristics of each site.

Table 4 shows that the Kalanjara Jadid, with a ratio of 0.6, experiences higher outdoor radiation levels. This is supported by its specific radionuclide activities, which show concentrations of 78.22 Bq/Kg for ^226^Ra, 114.38 Bq/Kg for 232Th, and 651.55 Bq/Kg for ^40^K, potentially contributing to the elevated outdoor gamma dose rate. In contrast, Tanda Banjara has a ratio of 1, indicating equal exposure levels indoors and outdoors, with radionuclide concentrations of 65.88 Bq/Kg for ^226^Ra, 98.21 Bq/Kg for ^232^Th, and 872.38 Bq/Kg for ^40^K. Rithaura Grunt, with a ratio of 1, also shows balanced indoor and outdoor exposure, with moderate levels of 74.86 Bq/Kg for ^226^Ra, 107.68 Bq/Kg for ^232^Th, and 420.37 Bq/Kg for^40^K. Dhandera (CT) presents a different scenario with a ratio of 1.4, suggesting higher indoor radiation levels. This location has the highest concentration of ^232^Th at 132.26 Bq/Kg, along with 89.43 Bq/Kg for ^226^Ra and 682.75 Bq/Kg for^40^K, which could be significant factors in the increased indoor gamma dose rate. Shahpur, on the other hand, has a ratio of 0.7, indicating higher outdoor exposure, with concentrations of 72.38 Bq/Kg for ^226^Ra, 86.87 Bq/Kg for ^232^Th, and 621.47 Bq/Kg for ^40^K. HakimPur Turra stands out with a ratio of 1.9, the highest in the dataset, suggesting significantly higher indoor radiation, with 67.84 Bq/Kg for ^226^Ra, 92.63 Bq/Kg for ^232^Th, and 421.33 Bq/Kg for ^40^K. Daluwala Khurd, with a ratio of 1.3, also experiences higher indoor exposure, supported by 68.46 Bq/Kg for ^226^Ra, 94.1 Bq/Kg for ^232^Th, and 320.18 Bq/Kg for ^40^K. Sikraudha, with a ratio of 1.1, indicates slightly higher indoor exposure, with balanced concentrations of 74.38 Bq/Kg for ^226^Ra, 109.12 Bq/Kg for ^232^Th, and 408.64 Bq/Kg for ^40^K. Mukarrabpur, showing a ratio of 1.3, has higher indoor exposure, with 65.28 Bq/Kg for ^226^Ra, 88.15 Bq/Kg for ^232^Th, and 420.66 Bq/Kg for ^40^K. Sahpur Salhapur, with a ratio of 0.9, indicates slightly higher outdoor exposure, supported by high ^40^K activity at 710.49 Bq/Kg, along with 59.12 Bq/Kg for ^226^Ra and 78.38 Bq/Kg for ^232^Th. Lastly, Daluwala Mazbata, with a ratio of 0.9, suggests higher outdoor exposure, with 74.65 Bq/Kg for ^226^Ra, 48.51 Bq/Kg for ^232^Th, and 468.25 Bq/Kg for ^40^K. These ratios, when analysed alongside the specific activities of radionuclides, provide valuable insights into the potential sources of radiation and the associated exposure risks in different environments.

**Fig. 5:**
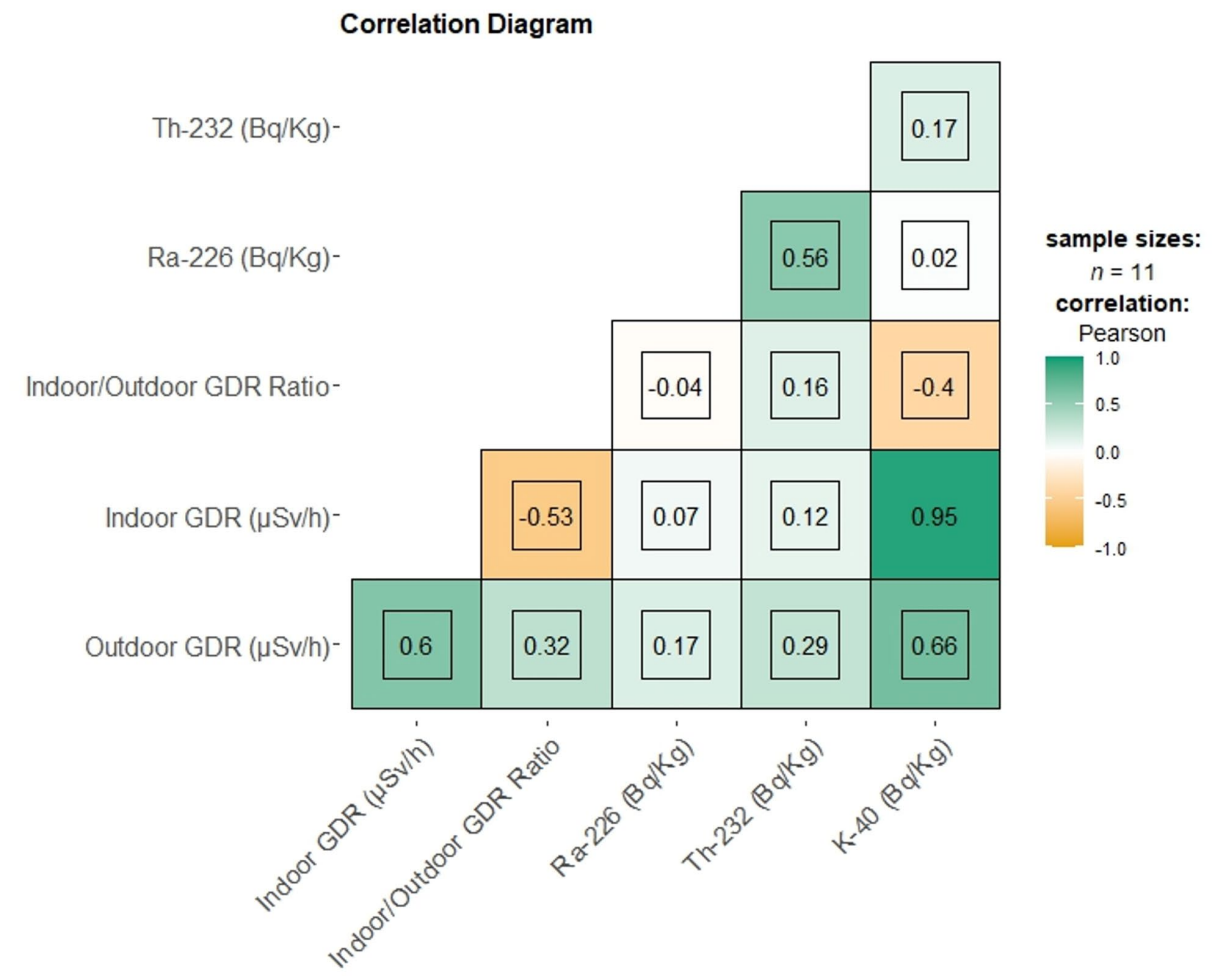
The diagram illustrating the Pearson correlation coefficients among various radiological parameters, including radionuclide concentrations (^226^Ra, ^232^Th, and ^40^K), gamma dose rates (indoor and outdoor), and indoor/outdoor ratio, with altitude.

Additionally, a correlation diagram has been generated for this obtained data of outliers to estimate the degree of their relationship to each other, which is depicted in Fig. 5. The correlation diagram reveals significant insights into the relationships among radiological and environmental parameters, particularly focusing on radionuclide concentrations (^226^Ra, ^232^Th, and ^40^K) and gamma dose rates (indoor and outdoor). A positive correlation (0.60) between indoor and outdoor gamma dose rates indicates a substantial influence of outdoor radiation on indoor environments. This suggests that outdoor radiation levels are a critical factor in determining indoor exposure, possibly due to the permeability of building materials to gamma radiation. The correlation between outdoor gamma dose rates and ^40^K concentration (0.66) highlights the role of ^40^K in contributing to elevated outdoor radiation levels, reflecting its natural abundance in geological formations.

The negative correlation between the indoor/outdoor GDR ratio and ^232^Th concentration (− 0.40) suggests that ^232^Th is more prevalent outdoors, potentially due to its association with specific rock types and soil compositions^44,45^. This finding implies that areas with higher ^232^Th concentrations may exhibit lower indoor/outdoor GDR ratios, indicating a disparity in radiation levels between environments. Additionally, the moderate positive correlation between outdoor gamma dose rates and ^226^Ra concentration (0.32) underscores the contribution of ^226^Ra to ambient radiation, reinforcing the impact of natural radionuclides on environmental radiation profiles. The inverse relationship between indoor gamma dose rates and the indoor/outdoor GDR ratio (− 0.53) suggests that higher indoor radiation levels are linked to lower ratios, potentially indicating inadequate shielding or the presence of indoor radiation sources. These results highlight the complex interactions between environmental factors and radiation exposure, emphasizing the need for targeted interventions to mitigate radiation risks and enhance public health protection.

## Conclusion

This study aimed to evaluate the variability of natural background radiation exposure from spontaneous gamma emissions from the terrestrial characteristics of the Haridwar district. Using a portable survey meter, the gamma dose rate (µSv/h) has been measured in an outdoor and indoor environment at 250 random locations within the administrative boundary of Haridwar. The dataset was analysed using statistical regression models and GIS techniques. Potential outliers were re-evaluated through radionuclide analysis of ^226^Ra, ^232^Th, and ^40^K in soil samples from these specific locations. The present study concludes in the following bullets:

1. *Indoor environments exhibit elevated gamma radiation levels compared to outdoor settings*: This discrepancy is likely attributed to building materials and reduced ventilation, which can lead to the accumulation of radon gas.
2. *Radiation exposure demonstrates an age-dependent variability*: Infants experience the highest radiation doses, underscoring the necessity of implementing age-specific radiation protection strategies.
3. *A complex interplay of environmental and structural factors governs gamma radiation levels*: Outdoor radiation is intricately linked to soil composition and geological characteristics, while indoor radiation is influenced by both external environmental conditions and the properties of building materials. The outliers are significantly explained by the specific activities of radionuclides (^226^Ra, ^232^Th, and^40^K) in soil samples taken from specific sites. Subsequently, there is a very high correlation between ^40^K and gamma dose rates, i.e., 66% with outdoor and 95% with indoor, has been observed in outliers. The results show the influence of local geological formations on the terrestrial gamma dose rate in the Haridwar.
4. *Average annual effective dose exceeds world average*: This survey concludes that the average annual effective dose in Haridwar is higher (almost double) than the world average of terrestrial background radiation, i.e., 0.48 mSv/y.
5. *Comprehensive investigations are warranted to elucidate outlier observations and terrain-specific variations*: It was found that the distributions of gamma dose rates are similar in both highland and lowland terrains. Unexpectedly high radiation readings in specific locations and subtle disparities between highland and lowland terrains necessitate further exploration to identify unmodeled factors that may contribute to radiation exposure patterns.
6. *Limitations of the study*: Gamma levels were measured at various outdoor locations during this study. Consequently, the results reflect exposure from the outdoor environment only. Indoor environments, where building materials are the primary radiation source, may present different net doses depending on specific conditions. However, statistical methods have inherent limitations as they provide average trends and generalize outcomes. Individual exposure levels can vary significantly.

## Data Availability

Data sets generated during the current study are available from the corresponding author on reasonable request.
